# Editorial

**Published:** 1875-11

**Authors:** 


### Editorial.

### OHIO STATE DENTAL SOCIETY.

The tenth annual meeting of this society will be held in the city of Columbus, beginning on the 1st day of Dec. next.

We would direct special attention to the official call on another page of this number. We doubt not, this will be one of the best meetings of this body ever held.

The programme is an excellent one and presents subjects for consideration of great importance, and we would suggest that every one make some preparation upon each, before the meeting; let none be satisfied with this, but bring everything new and valuable in a practical way. The meeting need not be confined to this programme, other subjects of interest may and probably will be introduced.

In view of the fact that this society has done so much for the profession in the state, and secured for it a status, not excelled, if equalled by the profession in any other state in the Union; it is proper and just that the profession should rally round its standard, and bring to it all moral and material support. This society should have a membership of at least three hundred; nine-tenths of whom should be present at every meeting, and then what grand meetings there would be and what an impetus the profession in the state would receive, every man would then receive an enthusiasm that would ripen into a holy zeal for the honor and welfare of his profession, guarding, defending and strengthening all its weak points. The committee on enrollment of the dentists of the state will have their report ready and it is desirable that every one be present to see that he has his true position on that list. Let there be a grand outpouring of the profession of the state, such as has never been seen before. There will doubtless be many new and valuable things to be seen and heard Come one! Come all!

### STATE BOARD OF EXAMINERS.

The Ohio State Board of Dental Examiners will be held in Columbus, at the American Hotel, on Wednesday, December 1st, prox at 12 m. It is desirable that all interested, report themselves promptly at that time so far as possible, that arrangements may be made for examinations that will not interfere with the sessions of the State Society.

### DENTAL VULCANITE CO.

The following communication presents the Dental Vulcanite Co. in a new role, one that the profession would do well to regard.

Such operations will serve to bring them, with all their agents, a greater measure of contempt than they have thus far enjoyed. Whatever may be the claims of that Co., wherein they have succeeded in obtaining injunctions, they should be, in the letter and spirit, obeyed. We do not believe that injunctions should be granted in this matter, nor do we believe they would if the case could have a hearing *de novo*, wholly free from the influence of former decisions, but that is now impossible and things must be accepted as they are and not as we might wish them.

Dr. B. in this instance was not led into the difficulty by the expectation of gain, but by the whining importunity of a hireling under a mask. But to the letter.

COLUMBUS, Oct. 25, '75.

DR. J. TAFT.

*Editor of Register:* I wish to state for the benefit of that portion of the readers of the REGISTER who are enjoined from the use of rubber for dental purposes, that Josiah Bacon has his spies in the field, and I would advise that they avoid the trap into which I was so unwittingly led. It was done on this wise. A man came into my office and said he wished two teeth inserted, I examined the mouth and found that it was the two superior bicuspids, the teeth opposite them in the lower jaw being gone, I was rather surprised that he should wish teeth in such a place, and especially as he was a very plain appearing man, poorly dressed and representing himself as a stone cutter, and very poor, he could not have gold plate because of expense, celluloid was suggested, he said he had tried that and the camphor made him sick, and rubber was the only thing that he thought he could wear, and if I would only make him this little piece it would be a great accommodation.

He also further stated, by way of inducement, that his mother wanted a full set and she would have them on celluloid, and would be in the next week but he must have his immediately.

Yielding to his importunity, I finally told my assistant to make it for him, as it was a small matter and there was no profit in it. The thing was done and the result was that upon this man's affidavit, I was summoned to appear before the district court for contempt, for this however I have not as yet been punished, and do not apprehend I shall be. This man went to nearly all the dentists in Columbus and had his impression taken, but mine was the first and only one finished before the true character of the man was found out. I have thus been sold and I would caution every one to be on the lookout. He was a small and not a prepossessing man.

The description I have given including that of his mouth, will identify him to any dentist. So be on the lookout for him or any such. Yours Respectfully, J. B. BEUAMAN.

---

### OHIO STATE DENTAL SOCIETY.

The tenth annual meeting of this society will be held in the City Council Chamber, at Columbus, on Wednesday, the 1st day of December, 1875, commencing at 10 o'clock a. m. and continuing its session three days.

C. R. BUTLER, President. J. M. PORTER Secretary.

ORDER OF BUSINESS.

1. Calling of the roll.
2. Payment of dues.
3. Reading of the minutes.
4. Reports of standing committees.
5. Reports of special committees.

Election of members at any convenient time during the session, upon report of committee.

Ehibition of instruments and appliances, from 2 to 3 o'clock p. m., second day.

Election of officers during the evening session of the second day.

Dental depots occupying rooms in the same building, in which the Society holds its meetings, will be politely requested to close their doors during the sessions.

SUBJECTS FOR DISCUSSION.

1. Diseases of the gums and alveoli. Causes and treatment.

2. Diseases of the maxillary sinus. Causes and treatment.

3. Reflex influences from diseased teeth. In what direction and to what extent do they occur?

4. Dental therapeutics.

5. Bleaching teeth.

6. Preparation of proximal cavities in bicuspids and molars, and filling the same with the different kinds of foil.

7. Miscellaneous subjects directly connected with mechanical dentistry.

Col. Blount, the landlord of the American Hotel, will entertain members or visitors to the Ohio State Dental Society, at two dollars per day.

A full attendance is respectfully urged upon the members, and every respectable practitioner of dentistry is most cordially invited to be present, and shall be made welcome as a visitor.

F. H. Rehwinkel, D. R. Jennings and J. H. Warner, Executive Committee.

THE STATE BOARD OF DENTAL EXAMINERS

Will hold a meeting at the American Hotel on Wednesday, the 1st day of December, 1875, at 12 o'clock, m. Candidates for examination will please take notice.

J. Taft, President of Board, W. P. Horton, Secretary.

### MERRIMAC VALLEY DENTAL ASSOCIATION.

The annual meeting of the above Society will be held in the Common Council Room, Manchester, N. H., on Thursday and Friday, Nov. 4th and 5th, '75 commencing at 10 a. m., Thursday.

ESSAYISTS.

Dr. D. G. Harrington, of Boston, Mass.; Dr. Abr. Robertson, of Georgetown, Mass.; Dr. A. P. Stevens, of Portsmouth, N. H. Volunteer essays may be expected from other members. Dr. T. Fillebrown, of Portland, Me., will give a Clinic showing the use of platinum foil.

SUBJECT FOR DISCUSSION.

"Operative and mechanical dentistry." A cordial invitation is extended to the profession, whether members of the Association or not, to meet with us. Hotel headquarters at the Haseltine House. W. E. Riggs, Secretary, Lawrence, Mass., Oct. 20, '75.

### MICHIGAN STATE DENTAL SOCIETY

Held its annual meeting at Ann Arbor, on the 12th, 13th and 14th of Oct. There was quite a large representation of the profession of Michigan present, and an excellent meeting was the result. The profession of this State is thoroughly alive to their best interests, and take hold of association work in real earnest, fully aware of its value and efficiency.

They feel that they have an enterprise worth working for in the Dental College established in connection with the University of Michigan, and if they continue their effort in this direction the Institution will be eminently a success. Several sessions of the meeting were held in the college building.

This association took quite an advanced position on the subject of dental education several years ago, one that has attracted the attention of the whole profession of the country and received its approbation.

The next meeting is to be held at the same place in the last week of March, next.

